# Real-life effectiveness and safety of vedolizumab in moderate-to-severe ulcerative colitis: A single-center experience in Northern China

**DOI:** 10.1097/MD.0000000000038759

**Published:** 2024-07-05

**Authors:** Jing Yan, Xueli Ding, Jun Wu, Ailing Liu, Liang Fang, Yonghong Xu

**Affiliations:** a Department of Gastroenterology, the Affiliated Hospital of Qingdao University, Qingdao, Shandong Province, China.

**Keywords:** effectiveness, loss of response, risk factor, safety, ulcerative colitis, vedolizumab

## Abstract

Vedolizumab (VDZ), a monoclonal antibody to α4β7 integrin, is available for patients with moderate-to-severe ulcerative colitis (UC). This study planned to assess the real-world effectiveness and safety of VDZ for UC patients in Northern China. We enrolled patients with moderate-to-severe UC who underwent VDZ induction therapy from March 2021 to November 2022 at the Affiliated Hospital of Qingdao University. The primary outcome was clinical remission at weeks 14 and 52 after the initial VDZ therapy. Overall adverse events and risk factors associated with loss of response (LOR) were also evaluated. Seventy-three UC patients receiving VDZ therapy were included in this study. The rates of clinical response, clinical remission, and steroid-free clinical remission were 69.9%, 39.7%, and 34.2% at week 14 and 90.5%, 66.7%, and 64.4% at week 52, respectively. The mucosal remission rates were 37.5% (18/48) at week 14 ± 8 and 27.3% (9/33) at week 52 ± 16, while only 2 and 3 patients achieved mucosal healing at weeks 14 ± 8 and 52 ± 16, respectively. Of the UC patients, 23.3% experienced adverse events associated with VDZ, most of which were mild and self-limiting. Until the last follow-up, 37 of 73 UC patients experienced LOR during the maintenance period. Patients with a higher ulcerative colitis endoscopic severity index (UCEIS), partial Mayo scores (PMS), or hemoglobin below 120 g/L at baseline were more likely to experience LOR after VDZ induction therapy. VDZ is an effective and safe agent for patients with moderate-to-severe UC in Northern China. A high baseline UCEIS, PMS, or hemoglobin < 120 g/L may be an independent risk factor for LOR during the maintenance period.

## 1. Introduction

Ulcerative colitis (UC) is a chronic recurrent disease characterized by persistent intestinal mucosal inflammation.^[1]^ In recent years, the incidence of UC has increased significantly in Asia, and acute severe UC has become a life-threatening challenge for patients.^[2,3]^ Traditional medications for UC include 5-aminosalicylate (5-ASA), steroids, and immunomodulators.^[4]^ 5-ASA is commonly used for induction and maintenance therapy in patients with mild UC but is often ineffective in moderate-to-severe UC. Corticosteroids and immunosuppressants are not suitable for long-term treatment in the maintenance phase because of their serious side effects.^[4,5]^ Fortunately, biological agents (such as infliximab [IFX], a tumor necrosis factor [TNF] inhibitor) have been shown to be effective in inducing and maintaining UC remission.^[6]^ But approximately 30% to 45% of patients, experience treatment failure due to primary or secondary treatment failure.^[7]^ Furthermore, apart from tuberculosis and other opportunistic infections, TNF inhibitors could increase the risk of non-melanoma skin cancer, which is further enhanced by the concomitant use of thiopurine therapy.^[8]^ Therefore, there is a need for novel medicines that can effectively induce and maintain clinical or endoscopic remission in patients with UC.

Known as “a human monoclonal antibody to the α4β7-integrin,” vedolizumab (VDZ) blocks lymphocyte trafficking to the intestine.^[9]^ Previous researches in the GEMINI trials demonstrated that VDZ was significantly more effective than placebo in the induction and maintenance of clinical remission and mucosal healing in patients who had received conventional therapy but still had active disease.^[10,11]^ VDZ may potentially be another choice for patients with moderate-to-severe UC who respond inadequately to conventional drugs or TNF inhibitors.^[12]^

Most research findings on the therapeutic response to VDZ in UC have been reported in Western populations with genetic and dietary habits different from those in Asian countries.^[13]^ Furthermore, a few variables impacting clinical results have been proposed, including a history of TNF exposure,^[14]^ first-line VDZ therapy,^[15]^ high baseline C-reactive protein (CRP),^[14]^ and IL-8 levels,^[16]^ but the results are inconsistent. In China, the incidence rate of UC has been increasing,^[17]^ and VDZ therapy for UC has been accepted into health insurance coverage for 1 year. Therefore, the use of VDZ for moderate-to-severe UC is increasing rapidly. Recently, a southern Chinese study has confirmed excellent clinical remission and mucosal healing rates of VDZ in UC patients, but no risk factor analysis of efficacy was performed.^[18]^ In contrast, there are few published data on the VDZ induction therapy in Chinese UC patients in the north, where the incidence of UC is much higher. We aimed to assess the effectiveness and safety of VDZ in real-world patients with UC in Northern China and identify risk factors associated with loss of response (LOR) during the maintenance period.

## 2. Materials and methods

### 2.1. Patients and treatment

We retrospectively enrolled UC patients undergoing VDZ therapy at the Department of Gastroenterology, Affiliated Hospital of Qingdao University (Qingdao, Shandong Province, China) from March 2021 to November 2022. The inclusion criteria were: clinical, radiological, endoscopic, and histological criteria for the diagnosis of UC; age ≥ 18 years at the time of VDZ treatment; moderate-to-severe UC prior to VDZ treatment (total Mayo score > 5); and completion of at least 3 doses of VDZ therapy in our hospital. Exclusion criteria included: age < 18 years; pregnancy or lactation; unclassified inflammatory bowel disease (IBD), Crohn disease, intestinal tuberculosis, and intestinal Behcet disease; and undetermined disease activity or no colonoscopy prior to treatment.

VDZ was infused either intermittently or on a scheduled basis. The scheduled regimen was defined as 3 infusions at weeks 0, 2 and 6, respectively, then regular infusions per 8 weeks. Intermittent treatment involved a shorter infusion interval (6 or 4 weeks) after regular induction therapy, rather than a fixed interval of every 8 weeks. All the patients received an intravenous dose of 300 mg each time.

### 2.2. Data collection

Demographic data, baseline characteristics, reasons for VDZ, prior and concomitant treatment, clinical and endoscopic outcomes, laboratory data (CRP, erythrocyte sedimentation rate [ESR], hemoglobin, albumin, leukocytes, and platelets), and adverse effects of VDZ therapy were collected from all qualified patients by browsing the electronic medical record system.

### 2.3. Definition

According to the Montreal classification, UC was classified as proctitis (E1), left-sided UC (E2), and total colitis (E3).^[19]^ Classification of disease activity as mild (3–5 points), moderate (6–10 points) and severe (11–12 points) was based on the total Mayo score, which includes stool frequency, rectal bleeding, Mayo endoscopy score (MES), and overall assessment by the physician. The partial Mayo score (PMS) was defined as the Mayo score without MES. The ulcerative colitis endoscopic index of severity (UCEIS) is the sum of several sub-scores for vascular patterns (0–2 points), hemorrhage (0–3 points), and erosions and ulcerations (0–3 points) in the most severe portion of mucosal inflammation.^[20]^ The definitions of steroid-refractory and steroid-dependent colitis were determined following the ECCO evidence-based consensus.^[21]^

### 2.4. Outcomes assessment

Clinical outcomes of VDZ were assessed by the PMS at baseline, weeks 14 and 52. Clinical response was defined as a decrease of at least 2 points from baseline in PMS (or at least 30% in PMS) accompanied by an absolute score for rectal bleeding of ≤ 1 or a decrease of at least one point from baseline. Clinical remission was considered as a PMS ≤ 2, with no sub-score above 1. Steroid-free clinical remission (SFCR) was defined as clinical remission with no combination of steroids. Mucosal remission and mucosal healing were defined as MES ≤ 1 and MES = 0, respectively. Additionally, the UCEIS score was used for further endoscopic evaluation. The changes in CRP, ESR, hemoglobin, albumin and leukocyte counts were also assessed and recorded. LOR to VDZ was defined as a clinical relapse requiring shortening of VDZ infusion interval or adding or switching to other alternative therapies (such as corticosteroids, tofacitinib, or other biological agents) among the responders during the maintenance period.

The primary endpoint was clinical remission at weeks 14 and 52. The secondary endpoints of this study included: clinical response and SFCR at weeks 14, and 52, respectively; mucosal remission and mucosal healing at weeks 14 ± 8 and 52 ± 16, respectively; LOR up to last follow-up.

### 2.5. Statistical analysis

Discrete variables were expressed as numbers and percentages; continuous variables with normal distribution or non-normal distribution were shown as mean ± standard deviation or median and interquartile range, respectively. The T-test or Mann-Whitney U-test was used to analyze continuous variables, and the chi-square test or Fisher exact test was used for discrete variables. Risk factors associated with LOR were assessed using Kaplan–Meier survival analysis and compared using the log-rank test. The SPSS software (version 26.0; SPSS Inc.) was used for statistical analysis, and graphs were generated using the GraphPad Prism (version 5.0). Statistical significance was set at a *P* value < .05.

### 2.6. Ethics approval and consent to participate

This study was approved by the ethics committee of the Affiliated Hospital of Qingdao University (NO. QYFY WZLL 27687). Consent to participate was waived by the ethics committee of the Affiliated Hospital of Qingdao University because this study was retrospective study.

## 3. Results

### 3.1. Patient characteristics

**Figure 1.**
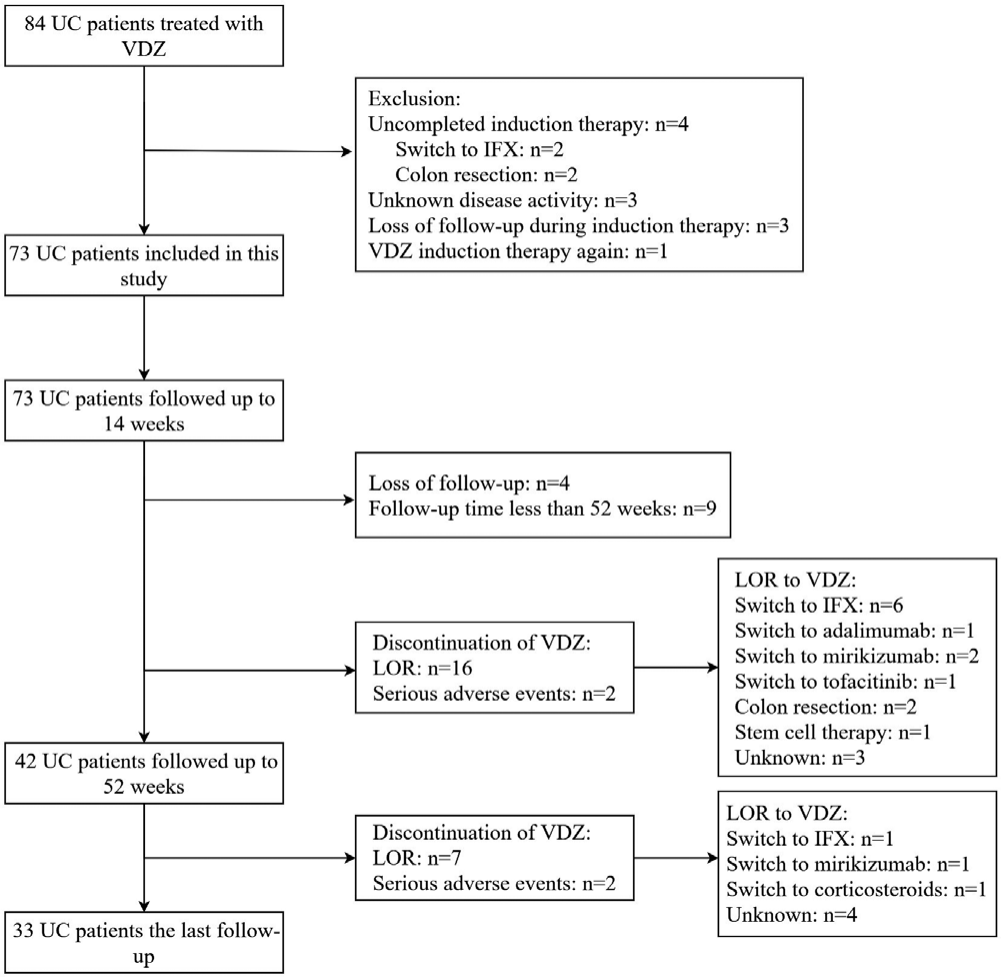
Flowchart of UC patients treated with VDZ. IFX = infliximab, LOR = loss of response, UC = ulcerative colitis, VDZ = vedolizumab.

A total of 73 patients with UC, including 50 males and 23 females, were included in this study (Fig. 1). The characteristics of all patients are shown in Table 1. The mean age at the first VDZ treatment was 47.2 ± 14.6 years (range 20–84 years) and the median disease duration was 5.0 (2.0, 9.5) years. The numbers of patients with total colitis (E3), left-sided colitis (E2) and proctitis (E1) were 55 (75.3%), 17 (23.3%), and 1 (1.4%), respectively. Thirteen (17.8%) patients had a history of smoking, with no family history in any of the patients. At the start of VDZ treatment, patients had a median total Mayo score of 10.0 (9.0, 11.0), PMS of 7.0 (6.0, 8.0), and UCEIS score of 6.0 (5.0, 8.0). Before VDZ infusion, all patients used 5-ASA, and 28 (38.4%), 10 (13.7%), and 6 (8.2%) patients received corticosteroids, anti-TNF agents, and immunosuppressants, respectively. With above treatments, 46 (63.0%) and 27 (37.0%) patients still had moderate and severe disease activity, respectively. Sixty-seven patients (91.8%) received concomitant drugs at the start of VDZ treatment, including 22 (30.1%) corticosteroids with 5-ASA, 1 (1.4%) corticosteroid, and 44 (60.3%) 5-ASA.

**Table 1:** Clinical characteristics of ulcerative colitis patients at baseline.

Characteristics	Total (N = 73)
Male gender, n (%)	50 (68.5)
Age at first VDZ infusion, years	47.2 ± 14.6
Disease duration, years	5.0 (2.0, 9.5)
BMI*, kg/m^2^	21.9 ± 2.8
Total Mayo score	10.0 (9.0, 11.0)
PMS	7.0 (6.0, 8.0)
History of smoking, n (%)	13 (17.8)
Extent of disease†, n (%)	
E1	1 (1.4)
E2	17 (23.3)
E3	55 (75.3)
Disease activity, n (%)	
Moderate	46 (63.0)
Severe	27 (37.0)
MES, n (%)	
Mayo 2	16 (21.9)
Mayo 3	57 (78.1)
UCEIS score	6.0 (5.0, 8.0)
Reason for VDZ therapy, n (%)	
Steroid-dependent	19 (26.0)
Steroid-refractory	6 (8.2)
Failure or intolerance of anti-TNF	10 (13.7)
Clinician/patient preference	38 (52.1)
Complication, n (%)	
Latent tuberculosis infections	3 (4.1)
Hepatitis B virus-related cirrhosis	1 (1.3)
Epstein-Barr virus infections	1 (1.3)
Previous medications, n (%)	
5-ASA	45 (61.6)
Corticosteroids + 5-ASA	28 (38.4)
Anti-TNF agents	10 (13.7)
Immunosuppressants	6 (8.2)
Concomitant medications, n (%)	
Corticosteroids + 5-ASA	22 (30.1)
Corticosteroids	1 (1.4)
5-ASA	44 (60.3)
CRP*, mg/L	6.1 (1.2, 12.8)
ESR*, mm/h	11.0 (7.0, 23.0)
Hemoglobin*, g/L	121.0 (102.0, 145.8)
Albumin*, g/L	37.5 (32.9, 41.8)
Leukocytes*, ×10^9^/L	7.0 (5.5, 9.1)
Platelets*, ×10^9^/L	272.5 (228.3, 361.0)

5-ASA = 5-aminosalicylate, BMI = body mass index, CRP = C-reactive protein, ESR = erythrocyte sedimentation rate, MES = Mayo endoscopy score, PMS = partial Mayo score, TNF = tumor necrosis factor, UCEIS = ulcerative colitis endoscopic severity index, VDZ = vedolizumab.

* BMI, CRP, ESR, hemoglobin, albumin, leukocytes and platelets were unknown in 72, 58, 59, 68, 67, 68, and 68, respectively.

† Diagnosis based on the Montreal classification; E1 proctitis, E2 left-sided UC, E3 total colitis.

The reasons for VDZ treatment were steroid-dependent colitis in 19 (26.0%), steroid-refractory colitis in 6 (8.2%), failure or intolerance to anti-TNF therapy in 10 (13.7%), and clinician/patient preference in 38 (52.1%) patients. Prior to VDZ therapy, 1 patient had postoperative breast cancer. Three (4.1%) patients had latent pulmonary tuberculosis and all received prophylactic treatment with anti-tuberculosis drugs. Additionally, 1 patient had hepatitis B virus-related cirrhosis and 1 patient had Epstein-Barr virus infection. Baseline laboratory data were as follows: CRP 6.1 (1.2, 12.8) mg/L, ESR 11.0 (7.0, 23.0) mm/h, hemoglobin 121.0 (102.0, 145.8) g/L, albumin 37.5 (32.9, 41.8) g/L, leukocytes 7.0 (5.5, 9.1) × 10^9^/L, and platelets 272.5 (228.3, 361.0) × 10^9^/L.

### 3.2. Clinical assessment

**Figure 2.**
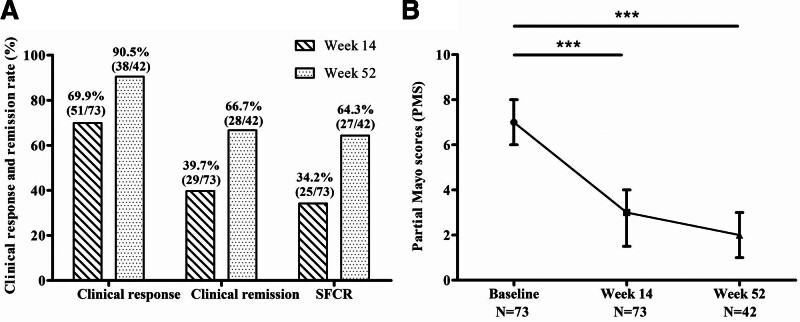
Clinical assessment of UC patients after VDZ therapy. (A) The rates of clinical response, clinical remission, and SFCR at weeks 14 and 52. (B) Partial Mayo score at baseline, weeks 14 and 52. ***P < .001. SFCR = steroid-free clinical remission, UC = ulcerative colitis, VDZ = vedolizumab.

The rates of clinical response, clinical remission, and SFCR, as well as the median PMS of UC patients after VDZ therapy, are shown in Figure 2. Of the 73 patients included in this study, 51 (69.9%) showed clinical response, 29 (39.7%) obtained clinical remission, and 25 (34.2%) achieved SFCR at week 14. Four patients who obtained clinical remission remained on concomitant steroids (Fig. 2A). The median PMS decreased from 7.0 (6.0, 8.0) at baseline to 3.0 (1.5, 4.0) at this time (Fig. 2B).

Of the 73 patients in this study, 9 were followed up for <52 weeks, 4 were lost after induction therapy, and the remaining 18 discontinued VDZ at week 52. Sixteen patients discontinued VDZ due to LOR except for 2 patients with severe side effects: 6 were switched to IFX, 1 to adalimumab, 2 to mirikizumab, 1 to tofacitinib, 2 to colon resection, 1 to stem cell therapy, and 3 were unknown (Fig. 1).

Forty-two patients were followed for 52 weeks after VDZ induction therapy, from which long-term efficacy data were obtained. 38 (90.5%) patients obtained clinical response, 28 (66.7%) were in clinical remission, and 27 (64.3%) were in SFCR, with a further decrease in the PMS to 2.0 (1.0, 3.0) (Fig. 2A and B).

### 3.3. Endoscopic and laboratory assessment

**Figure 3.**
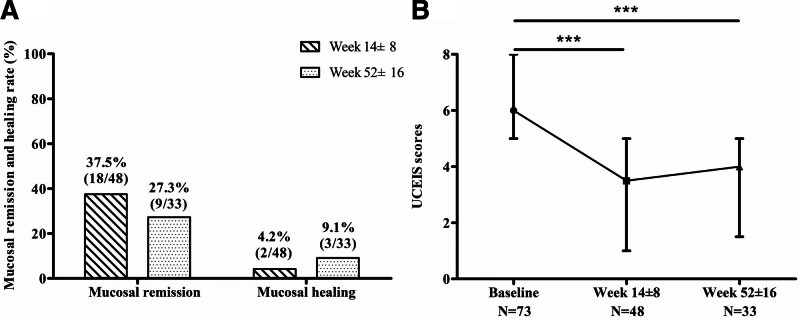
Endoscopic assessment of UC patients after VDZ therapy. (A) The rates of mucosal remission and mucosal healing at weeks 14 ± 8 and 52 ± 16. (B) UCEIS score at baseline, weeks 14 ± 8 and 52 ± 16. ***P < .001. UC = ulcerative colitis, UCEIS = ulcerative colitis endoscopic severity index, VDZ = vedolizumab.

Forty-eight and thirty-three patients underwent colonoscopy at 14 ± 8 and 52 ± 16 weeks, respectively (Fig. 3). At week 14 ± 8, 18 patients (37.5%) obtained mucosal remission, of which 2 (4.2%) obtained mucosal healing. Of the 33 patients who underwent colonoscopy at week 52 ± 16, 9 (27.3%) obtained mucosal remission, and 3 (9.1%) obtained mucosal healing (Fig. 3A). The median UCEIS score improved from 6.0 (5.0, 8.0) at baseline to 3.5 (1.0, 5.0) at week 14 ± 8, and to 4.0 (1.5, 5.0) at week 52 ± 16 (Fig. 3B).

**Figure 4.**
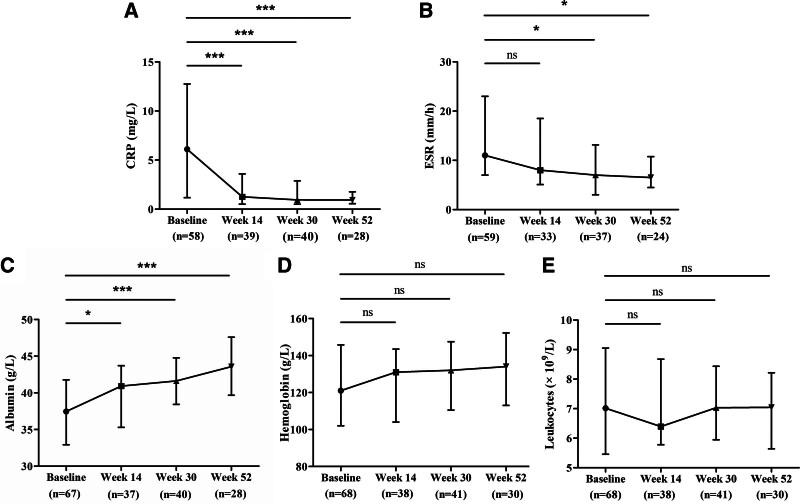
Effect of VDZ on CRP (A), ESR (B), albumin (C), hemoglobin (D), and leukocytes (E) at baseline, wk 14, 30, and 52. ns *P* > .05; *P < .05, **P < .01, ***P < .001. CRP = C-reactive protein, ESR = erythrocyte sedimentation rate, VDZ = vedolizumab.

A significant decrease in CRP levels and an increase in albumin levels were noted at weeks 14, 30, and 52 of VDZ therapy in comparison to baseline. In addition, ESR decreased significantly from baseline to weeks 30 and 52 after VDZ therapy. Although anemia and hyperleukocytosis of these patients were corrected at weeks 14, 30, and 52 after therapy, no significant improvement was observed (Fig. 4).

### 3.4. Assessment of LOR

The maximum follow-up period was 116 weeks. Until the last follow-up, 37 of 73 UC patients experienced LOR during the maintenance period. Eight patients shortened the infusion interval of VDZ (1 to 4 weeks and 7 to 6 weeks), 16 switched to other therapies (1 to corticosteroids, 7 to IFX, 1 to adalimumab, 3 to mirikizumab, 1 to tofacitinib, 2 to colon resection, and 1 to stem cell therapy), 6 added other therapies (4 with corticosteroids and 2 with tofacitinib), and 7 were unknown but discontinued VDZ (Table 2).

**Table 2:** Ulcerative colitis patients with LOR until the last follow-up.

		N = 37
Shortening of VDZ infusion interval	4 wk	1
	6 wk	7
Switching to other therapies	Corticosteroids	1
	IFX	7
	Adalimumab	1
	Mirikizumab	3
	Tofacitinib	1
	Colon resection	2
	Stem cell therapy	1
Adding other therapies	Corticosteroids	4
	Tofacitinib	2
Unknown		7

IFX = infliximab, LOR = loss of response, VDZ = vedolizumab.

### 3.5. Risk factors for LOR in UC

In the univariate analysis with log-rank test, patients with PMS ≥ 7 (χ^2^ = 4.587, *P* = .032), UCEIS score ≥ 7 (χ^2^ = 4.965, *P* = .026), and serum hemoglobin < 120 g/L (χ^2^ = 6.724, *P* = .010) had a significantly higher likelihood of LOR. However, no differences were found in the percentage of LOR with respect to gender (χ^2^ = 0.188, *P* = .664), age at first VDZ infusion (χ^2^ = 0.089, *P* = .765), disease duration (χ^2^ = 0.068, *P* = .794), BMI(χ^2^ = 0.055, *P* = .815), history of smoking (χ^2^ = 0.000, *P* = .995), extent of disease (E1/E2/E3: χ^2^ = 0.325, *P* = .568; χ^2^ = 1.162, *P* = .281; χ^2^ = 0.884, *P* = .347), MES (χ^2^ = 0.489, *P* = .485), steroid-dependent colitis (χ^2^ = 0.147, *P* = .701), steroid-refractory colitis (χ^2^ = 1.472, *P* = .225), previous medications (corticosteroids + 5-ASA/anti-TNF agents/immunosuppressants: χ^2^ = 1.934, *P* = .164; χ^2^ = 0.793, *P* = .373; χ^2^ = 0.020, *P* = .888), concomitant medications (corticosteroids + 5-ASA/corticosteroids/5-ASA: χ^2^ = 0.000, *P* = .993; χ^2^ = 0.557, *P* = .455; χ^2^ = 0.047, *P* = .829), CRP (χ^2^ = 0.023, *P* = .879), ESR (χ^2^ = 1.074, *P* = .300), albumin (χ^2^ = 0.140, *P* = .709) and platelet count (χ^2^ = 0.849, *P* = .357) (Table 3).

**Table 3:** Risk factors associated with LOR by univariate analysis.

Variables	LOR, n/N(%)	*χ* ^2^	*P* value (log-rank)	95%CI
Gender		0.188	.664	
Male	25/50 (50.0)			44.2–73.9
Female	12/23 (52.2)			36.7–73.3
Age at first VDZ infusion (yr)		0.089	.765	
<60	31/57 (54.4)			41.4–68.3
≥60	6/16 (37.5)			26.8–95.2
Disease duration (yr)		0.068	.794	
≤5	22/40 (55.0)			26.3–71.7
>5	15/33 (45.5)			52.5–65.5
BMI (kg/m^2^)		0.055	.815	
≥24	11/21 (52.4)			57.5–64.5
<24	26/51 (51.0)			36.6–72.4
History of smoking		0.000	.995	
Yes	7/13 (53.8)			35.0–74.0
No	30/60 (50.0)			51.1–70.9
Extent of disease				
E1	0/1 (0)	0.325	.568	NA
E2	11/17 (64.7)	1.162	.281	34.4–75.6
E3	26/55 (47.3)	0.884	.347	NA
PMS		4.587	**.032**	
<7	9/25 (36.0)			NA
≥7	28/48 (58.3)			38.6–70.4
MES		0.489	.485	
Mayo 2	7/16 (43.8)			57.0–65.0
Mayo 3	30/57 (52.6)			34.8–74.2
UCEIS score		4.965	**.026**	
<7	18/44 (40.9)			NA
≥7	19/29 (65.5)			26.4–57.6
Steroid-dependent colitis		0.147	.701	
Yes	10/19 (52.6)			NA
No	27/54 (50.0)			51.0–67.1
Steroid-refractory colitis		1.472	.225	
Yes	5/6 (83.3)			0.6–108.4
No	32/67 (47.8)			46.6–75.4
Previous medications				
Corticosteroids + 5-ASA	17/28 (60.7)	1.934	.164	9.2–58.8
Anti-TNF agents	4/10 (40.0)	0.793	.373	NA
Immunosuppressants	3/6 (50.0)	0.020	.888	NA
Concomitant medications				
Corticosteroids + 5-ASA	12/22 (54.5)	0.000	.993	30.6–79.4
Corticosteroids	0/1 (0)	0.557	.455	NA
5-ASA	22/44 (50.0)	0.047	.829	40.2–81.8
CRP (g/L)		0.023	.879	
≤5	13/27 (48.1)			36.7–72.3
>5	16/31 (51.6)			33.5–76.5
ESR (mm/h)		1.074	.300	
≤15	18/38 (47.4)			44.6–79.4
>15	11/21 (52.4)			53.7–56.3
Hemoglobin (g/L)		6.724	**.010**	
≥120	15/37 (40.5)			NA
<120	20/31 (64.5)			27.8–56.2
Albumin (g/L)		0.140	.709	
≥35	22/43 (51.2)			47.4–74.6
<35	13/24 (54.2)			33.5–75.5
Platelet count (×10^9^/L)		0.849	.357	
≤300	21/43 (48.8)			51.2–70.8
>300	14/25 (56.0)			10.7–75.4

5-ASA = 5-aminosalicylate, BMI = body mass index, CRP = C-reactive protein, ESR = erythrocyte sedimentation rate, LOR = loss of response, MES = Mayo endoscopy score, PMS = partial Mayo score, TNF = tumor necrosis factor, UCEIS = ulcerative colitis endoscopic severity index, VDZ = vedolizumab.

### 3.6. Safety

Seventeen patients (23.3%) underwent adverse effects on the duration of VDZ treatment (Table 4). Of these, arthralgia (6/17, 35.3%) was the most common, followed by elevated liver transaminase level (4/17, 23.5%). Other adverse events included fever (2/17, 11.8%) and allergic reaction (1/17, 5.9%). Only 4 patients discontinued VDZ treatment due to anti-neutrophil cytoplasmic antibodies (ANCA)-associated vasculitis, eosinophilia, interstitial pneumonia, and laryngeal leukoplakia, respectively. However, most of the adverse effects were tolerable and mild. Three patients (4.1%) with combined pulmonary tuberculosis did not suffer reactivation of this disease during the follow-up period.

**Table 4:** Adverse events during VDZ treatment.

Total adverse events	N = 17
Arthralgia	6
Abnormal liver function	4
Allergic reaction	1
Fever after infusion	2
ANCA-associated vasculitis	1
Eosinophilia	1
Interstitial pneumonia	1
Laryngeal leukoplakia	1

ANCA = anti-neutrophil cytoplasmic antibodies, VDZ = vedolizumab.

## 4. Discussion

In recent years, as the incidence of UC has risen dramatically around the world, moderate-to-severe UC has become a critical challenge affecting patients’ life expectancy.^[1–3]^ VDZ may be another alternative therapy for patients with moderate-to-severe UC, including those who have lost response to conventional drugs or anti-TNF agents.^[9]^ Six studies^[22–27]^ in non-Asian countries evaluated the efficacy of VDZ at 6 to 14 weeks, with clinical response proportions of 58% to 75% and clinical remission proportions of 31% to 56%. With regard to long-term efficacy, the proportions of clinical response were 52% to 75% and clinical remission were 38% to 61% at 6 to 12 months.^[22–25,27]^ However, the genetic profile of Asian UC patients is different from that of Western populations.^[13]^ Only a few studies from Asian countries have reported the efficacy and safety of VDZ in the real-world. A Korean study suggested that 68.0% and 44.0% of UC patients who had failed previous anti-TNF agents obtained clinical response and remission at week 14, respectively, and 32.4% of patients were in mucosal remission at this point after VDZ therapy.^[28]^ Recently, a study by Huang et al^[18]^ in southern China found that the clinical remission rates of VDZ in patients with moderate-to-severe UC at weeks 14 and 52 were 65.6% and 64.1%, respectively, and the mucosal healing rates at weeks 14 ± 8 and 52 ± 8 were 38.2% and 35.3%, respectively. However, their study lacked risk factors analysis of clinical outcomes. To our knowledge, there are few published data on UC patients in Northern China receiving VDZ-induced therapy.

In our study, the proportions of clinical response and clinical remission were 69.9% and 39.7% at week 14, and 90.5% and 66.7% at week 52, respectively. These results were comparable to 2 studies conducted in Taiwan, China. The clinical response and clinical remission rates were 56.8% and 32.4%, respectively, at weeks 8 to 10 in the study by Kuo et al^[29]^ and 76.0% and 58.0%, respectively, at week 51 in Lin et al’ study.^[30]^ Moreover, we found that mucosal remission was achieved in 37.5% (18/48) patients at week 14 ± 8, which was similar to the findings of Kim et al who reported a mucosal remission rate of 32.4% (week 14). However, our study had a lower clinical remission rate at week 14 than the study by Huang et al (39.7% vs 65.6%), with a similar clinical remission rate of 64.1% at week 52. In addition, the rates of mucosal remission and healing were lower than those reported in their study (mucosal remission: 37.5% vs 64.7% at week 14; 27.3% vs 70.6% at week 52; mucosal healing: 4.2% vs 38.2% at week 14; 9.1% vs 35.3% at week 52). Notably, the percentage of patients with severe UC included in our study was 37.0%, which was higher than that in the study by Huang et al (20.3%). And it has been shown that more severe disease activity in VDZ therapy negatively affects patients’ clinical remission.^[31]^ Furthermore, the inflammation-related indexes such as CRP and ESR of UC patients after VDZ treatment decreased significantly from baseline, while serum albumin increased significantly from baseline, indicating that the systemic inflammatory activity and nutritional status of patients were improved.

Regarding the safety of VDZ, the incidence of adverse events in this study was 23.3%, which was slightly lower than the study by Huang et al (29.7%).^[18]^ The most common adverse event was arthralgia followed by abnormal liver function. These adverse events were contained in the VDZ Global Safety Database.^[32]^ Abbenante et al^[33]^ reported previously a case of UC patient in whom nodular vasculitis was detected during VDZ treatment. Similarly, 1 patient in this study was discovered with vasculitis during treatment, which was confirmed to be ANCA-associated vasculitis by pathologic biopsy and laboratory tests. Although 4 patients discontinued their VDZ infusions due to serious adverse events, none were life-threatening, and no cancer was found. Additionally, as reported by Ng et al,^[34]^ VDZ did not increase the risk of reactivation of tuberculosis infections, which is consistent with the findings of this study. VDZ therapy is a reliable option for these patients with tuberculosis infections.

LOR has become an essential clinical challenge in UC patients treated with VDZ therapy.^[35]^ The mechanism of LOR to VDZ remains unclear, and the relationship between pharmacokinetics and LOR has not been identified.^[36]^ In the report by Shmidt et al,^[37]^ the cumulative rate of LOR to VDZ in UC patients was 18% and 39% at months 6 and 12, and higher baseline CRP was a significant predictor of LOR. Univariate analysis was performed in this study to explore the risk factors associated with LOR, and we found that the UCEIS, PMS, and hemoglobin at baseline were related to LOR in UC patients after receiving VDZ induction therapy.

In 2012, Travis et al^[20]^ developed a novel evaluation tool for endoscopic disease severity covering vascular patterns, bleeding, and erosions/ulcers, known as the UCEIS. Di Ruscio et al^[38]^ found that a UCEIS score ≥ 7 at baseline but not MES was associated with no-response to biological agents and the requirement for colectomy in UC patients, but only 3 patients treated with VDZ were involved in their study. Our study found that UC patients with baseline UCEIS scores ≥ 7 were more likely to undergo LOR during the VDZ maintenance treatment period than those with baseline UCEIS scores < 7 (65.5% vs 40.9%). However, there was no difference in the LOR rates between MES 2 and MES 3. This discrepancy is probably since the UCEIS score includes an accurate description of the vascular patterns, bleeding, and erosions/ulcers compared to the MES. As recognized by Ikeya et al,^[39]^ the UCEIS was superior to MES in distinguishing between deep and shallow ulcers. Therefore, endoscopists need to pay attention to UCEIS scores in addition to MES in the management of UC patients undergoing VDZ treatment.

In the treatment of IBD, clinical disease severity at baseline is strongly associated with the choice of treatment regimen and treatment prognosis. Kotze et al^[31]^ discovered that UC patients with a PMS of ≥ 5 were less likely to achieve clinical remission at multiple time points (3, 6, and 12 months) after VDZ. In contrast, this study defined the treatment outcome of predictors analysis as LOR to VDZ. From this perspective, we found that a baseline PMS of ≥ 7 may be a risk factor for LOR in UC patients receiving VDZ. Patients with a lower baseline PMS had a significantly lower rate of LOR than those with higher scores (40.9% vs 65.5%). Therefore, clinicians may consider intensive therapy for UC patients with high baseline PMS to achieve clinical remission earlier.

Previous studies have confirmed that inflammatory anemia negatively affects the prognosis of treatment for patients with IBD.^[40]^ In a retrospective study of 35 of 75 IBD patients with anemia, Scarozza et al^[41]^ found a strong correlation between correction of anemia and clinical response to VDZ. Clinical responses were achieved in 91% of patients who had anemia correction but in only 45% of patients whose anemia did not improve. Given that Scarozza et al^[41]^ have demonstrated a positive effect of VDZ in UC patients with anemia, our analysis focuses more on the correlation between hemoglobin and LOR during the maintenance phase of VDZ. We found that 2-thirds of UC patients with hemoglobin < 120 g/L at baseline developed LOR after VDZ induction therapy, which was significantly higher than in patients with baseline hemoglobin ≥ 120 g/L. Hemoglobin used as one of the key parameters in the Truelove-Witts criteria,^[42]^ is closely related to disease activity, since both anemia and blood loss are caused by intestinal inflammation.^[40]^ Our study confirmed the correlation between indicators reflecting disease severity (PMS and UCEIS) and treatment outcome, while baseline hemoglobin level as a predictor of LOR was parallel to this result.

There are several limitations in this study. First, the small size of this study population and the missing values of hemoglobin at baseline did not allow a multivariate analysis to be performed after univariate analysis. Therefore, a multicenter study with a large cohort is needed for prospective evaluation to further validate risk factors affecting the prognosis of VDZ in clinical practice. Second, the points in time of endoscopic assessment are not uniform due to the individual willingness of patients with UC. The endoscopic results were analyzed at weeks 14 ± 8 and 52 ± 16 after VDZ treatment. Third, information about fecal calprotectin and VDZ trough levels, which have been shown to be correlated with prognosis in UC patients, was not available.^[43,44]^

In conclusion, we demonstrated that VDZ has excellent clinical effectiveness and safety for patients with moderate-to-severe UC in Northern China. Patients with a higher UCEIS, PMS, or hemoglobin below 120 g/L at baseline were more likely to experience LOR after VDZ induction therapy. Risk factors of LOR will help guide clinicians in treatment and improve outcomes.

## Author contributions

**Conceptualization:** Jing Yan, Xueli Ding.

**Data curation:** Xueli Ding, Ailing Liu.

**Formal analysis:** Jing Yan, Jun Wu, Yonghong Xu.

**Funding acquisition:** Ailing Liu.

**Investigation:** Jun Wu, Ailing Liu, Liang Fang.

**Methodology:** Jing Yan, Xueli Ding, Yonghong Xu.

**Project administration:** Xueli Ding, Jun Wu.

**Resources:** Jun Wu.

**Software:** Xueli Ding, Liang Fang.

**Supervision:** Jun Wu, Liang Fang.

**Validation:** Liang Fang, Yonghong Xu.

**Visualization:** Jing Yan, Ailing Liu.

**Writing – original draft:** Jing Yan.

**Writing – review & editing:** Yonghong Xu.
